# Infection dynamics of *Cryptosporidium bovis* and *Cryptosporidium ryanae* in a Swedish dairy herd

**DOI:** 10.1016/j.vpoa.2019.100010

**Published:** 2019-04-07

**Authors:** Malin Åberg, Ulf Emanuelson, Karin Troell, Camilla Björkman

**Affiliations:** a Department of Clinical Sciences, Swedish University of Agricultural Sciences, Box 7054, SE-750 07 Uppsala, Sweden; b Department of Microbiology, National Veterinary Institute, SE-751 89 Uppsala, Sweden

**Keywords:** Cryptosporidiosis, *Cryptosporidium* spp., Cattle, Pre-weaned calves, Diarrhea

## Abstract

In order to investigate the infection dynamics of *Cryptosporidium bovis* and *Cryptosporidium ryanae*, a Swedish dairy farm known to be free of *C. parvum* was recruited for a one-year study. Fecal samples were collected every four weeks (a total of 13 occasions) from 20 randomly selected calves younger than 65 days. A total of 238 feces samples were collected directly from the rectum and then examined with immunofluorescence microscopy; those positive for *Cryptosporidium* spp. oocysts were further processed by molecular species determination. Oocysts were found in 92 samples, and in 72 of these the species was successfully determined: 87.5% *C. bovis,* 9.7% *C. ryanae* and 2.8% a mix of both species. No *C. parvum* was found. The prevalence of shedding calves was highest at ages 4 and 5 weeks; however, the feces with the highest oocyst per gram (OPG) were registered at 2–4 weeks of age. This is earlier than what has been found in most other studies, indicating that the infection dynamics of *C. bovis/C. ryanae* may be affected by the presence of *C. parvum*. The OPG for the positive calves ranged from 200 to 1.1 × 10^6^ (geometric mean 6.88 × 10^3^). The youngest calf in which *C. bovis* was identified was 5 days old, and the youngest calf in which *C. ryanae* was identified was 15 days old. Calves’ housing type and seasonality were not associated with differences in the shedding of oocysts. Furthermore, there was no association between the presence of diarrhea and oocyst shedding.

## Highlights

• Both *C. bovis* and *C. ryanae*, but no *C. parvum,* were found in the pre-weaned calves.
• The earliest confirmed *C. bovis* oocyst shedding was observed in a 5-day-old calf.
• The shedding of *C. bovis* and *C. ryanae* was not associated with diarrhea.

## 1 Introduction

*Cryptosporidium* spp. are protozoan parasites that are found worldwide, infecting humans and all classes of animals. The parasites are transmitted through the fecal-oral route either by direct transmission from individual to individual or by consumption of contaminated food or water. Depending on the species of *Cryptosporidium* and its pathogenicity to the host, exposure may result in an asymptomatic infection or may cause illness, such as malnutrition, watery diarrhea, and fever (Fayer et al., 2009). In cattle, the first case of cryptosporidiosis was described in a heifer in 1971 (Panciera et al., 1971). Since then, *Cryptosporidium* spp. are a common finding on farms, both with and without calf diarrheal problems (Graczyk et al., 1997).

Four species are found on a regular basis in cattle: *C. parvum, C. bovis, C. ryanae* and *C. andersoni. C. parvum* infects the intestines and causes diarrhea in a wide range of hosts, including cattle and humans (Ryan and Hijjawi, 2015). The other three species have a narrower range of hosts and are mainly found in cattle. *C. bovis* was first described as a new genotype (*Cryptosporidium* genotype Bovine B) in cattle in 2002 (Xiao et al., 2002) and was recognized as a species three years later (Fayer et al., 2005). Unlike *C. parvum, C. bovis* has not been associated with clinical diarrhea in cattle. *C. ryanae* (previously *Cryptosporidium* deer-like genotype) is often found with *C. bovis* in post-weaned calves (Fayer et al., 2008). The oocysts of *C. parvum, C. bovis*, and *C. ryanae* are similar in size and shape, and molecular methods must be used to determine the species (Fayer et al., 2005, Fayer et al., 2008). *C. andersoni* is larger in size and infects the abomasum of juvenile and adult animals (Peng et al., 2003).

In most studies from around the world, *C. parvum* is the predominant species infecting pre-weaned calves (Fayer et al., 2010, Featherstone et al., 2010, Garro et al., 2016), whereas *C. bovis* and *C. ryanae* are mostly found in post-weaned calves (Fayer et al., 2010). In Sweden, however, reports show a different infection pattern, with *C. bovis* predominating among pre-weaned calves (Silverlås et al., 2010a, Silverlås et al., 2010b, Silverlås and Blanco-Penedo, 2013, Björkman et al., 2015). A few studies—from China, Canada and India—also report a dominance of *C. bovis* in pre-weaned calves (Feng et al., 2007, Wang et al., 2011, Budu-Amoako et al., 2012, Fan et al., 2017). In one Swedish study, samples from diarrheic calves had high fecal counts of oocysts per gram (OPG) of *C. bovis*, and no *C. parvum* or other diarrheal agents were found (Silverlås et al., 2010a, Silverlås et al., 2010b). This made the authors question the presumption that *C. bovis* is apathogenic to calves and suggested that it may cause illness under certain circumstances.

To our knowledge, there has been no longitudinal study of pre-weaned calves on a farm where only *C. bovis* and *C. ryanae* are present. To investigate the infection dynamics of these *Cryptosporidium* species, a Swedish dairy farm known to be free of *C. parvum* was recruited for a one-year study.

## 2 Material and methods

The study was approved by the Regional Ethical Review Board in Uppsala, Sweden (reference number C299/11).

### 2.1 Animals and housing

Animals on the designated farm had previously been sampled several times over the years for different projects, with findings of *C. bovis,* but never any *C. parvum*. The farm was declared free of Bovine viral diarrhea virus and *Salmonella* spp.

The farm was an organic dairy farm with approximately 140 cows and two automatic milking stations. Cows were of the Swedish Red breed and were held in a loose housing system with resting cubicles lined with rubber mats. Animals were kept on pasture in the summer months. Cows nearing calving were routinely moved to a group pen with deep straw bedding, except in summer when they were calving outdoors on pasture.

Newborn calves stayed with their dams to suckle for one day before they were moved to single pens and given additional colostrum. The single pens had slatted floors with straw bedding. Calves could make physical contact with their neighbors as per Swedish regulations. After approximately two weeks, the calves were subsequently merged into groups of five or six, in either indoor pens with rubber mats and woodchips or outdoor calf huts with deep straw bedding over concrete. They were fed whole milk until weaning at 12 weeks. They had free access to hay and were gradually introduced to concentrate.

While most male calves older than 4 months were sold, female calves were moved to an indoor group pen with resting cubicles with rubber mats and woodchips. When they were approximately 12–13 months old they were inseminated and moved to a group housing for heifers. Some of the heifers older than four months were sent to a grower until a couple of months before calving. The heifers that were nearing calving were moved to a separate section in the cow barn.

### 2.2 Bio-security

The farm was not closed to visitors, but anybody visiting the barns had to disinfect their boots with Vircon® and take appropriate precautions, such as hand washing, to prevent the spread of disease. Most cows had been raised from the farm’s own heifers, but to meet the needs of his expanding herd the farmer purchased some adult milking cows. As mentioned above, some heifers were housed at a neighboring farm for about a year. A few of the farm's cows had been shown at exhibition fairs.

### 2.3 Pre-study

To determine the presence of *Cryptosporidium* spp. on this farm, a first visit was made in January 2012 to collect a total of 50 fecal samples from cattle of all age categories. All the calves in single pens (*n* = 5) were sampled. Random samples from 17 pre-weaned calves and 9 young heifers (5–6 months) were taken. An additional 9 two-year-old heifers and 10 cows were convenience sampled.

### 2.4 Cross-sectional study

The repeated cross-sectional study began in June 2012 and ended in late April 2013. Sampling was conducted every four weeks, for a total of 13 occasions. At each occasion, 20 calves up to 65 days old were selected randomly before sampling. If there were fewer than 20 individuals in this age group, all were sampled. If the age criterion was met, an individual could be sampled on two separate occasions.

Fecal samples were collected directly from the rectum, and the fecal consistency was recorded according to four grades: firm, pasty, loose, and diarrheic. The specimens were kept at 4 °C until processing.

The body condition of the calves (thin, normal, or fat) and any ailment such as coughing or lameness were noted. The pen bedding was assessed for the amount of feces and straw or woodchips, and whether it was wet, damp, or dry.

### 2.5 Flotation method

One gram of each fecal sample was cleaned using a saline-glucose flotation method, as described by Maddox-Hyttel et al. (2006). Cleaned samples were stored at 4 °C until microscopy was performed and DNA extracted.

### 2.6 Microscopy

Ten microliters of cleaned sample were transferred to a 12 mm Ø well of a 3-well Teflon printed microscope slide (*Immuno-Cell, Mechelen, Belgium*), together with 50 μl distilled water. The slides were left to dry and then fixated in acetone for 5–10 min. The fixated samples were stained with 20 μl FITC-labelled anti-*Cryptosporidium* monoclonal antibodies (Crypto Cel, Cellabs, Australia) for one hour. The wells were then washed 3 times with 100 microliters of PBS. Ten microliters of mounting fluid were added to each well, and the entire well area was examined by epifluorescence microscopy at 200x and 400x magnification. The detection limit was 1 oocyst/10 μL sample, which translates into 200 oocysts per gram feces (OPG).

### 2.7 DNA extraction, gene amplification, and sequencing

All samples that were positive for *Cryptosporidium* spp. by microscopy were further analyzed at the 18S rRNA to determine the species. To verify the absence of *C. parvum,* all positive specimens were also analyzed at the 60 kDa glycoprotein (gp60) gene, which is found in *C. parvum.*

DNA was extracted from concentrated samples using a combined freeze-thawing and QIAamp DNA stool mini kit (QIAGEN, Hilden, Germany) protocol (Silverlås et al., 2010a, Silverlås et al., 2010b). Samples were then subjected to PCR protocols to amplify the 18S and gp60 genes. Amplification of the 18S gene was done essentially as described by Xiao et al. (1999). The first amplification mixture contained 1X of Platinum Taq buffer (Life Technologies), 3 or 4 mM MgCl_2_, 200 μm each of deoxynucleoside triphosphate, 200 μm each of primary primers, 0.16 mg/ml bovine serum albumin (BSA), 2 μl DNA solution, and 1 unit of Platinum Taq polymerase in a total volume of 25 μl. After initial denaturation at 95 °C for 5 min, 35 cycles followed, consisting of 94 °C for 45 s, 56 °C for 45 s, 72 °C for 60 s, and a final extension of 72 °C for 7 min. For the second amplification, 2 μl from the first reaction were added to a reaction mixture as above, except containing secondary primers and MgCl_2_ at 3 mM concentration. The reaction condition was 95 °C for 5 min, followed by 40 cycles of 94 °C for 45 s, 58 °C for 90 s, 72 °C for 60 s, and then a final extension step of 72 °C for 7 min. Amplification of the gp60 gene was done according to Chalmers et al. (2005).

Species were determined by sequencing in both directions on an ABI 3100 Genetic Analyzer (Applied Biosystems) using the internal primers and BigDye Terminator v3.1 Cycle Sequencing Kit (Applied Biosystems). Sequence data was assembled manually in BioEdit v7.0.5 sequence alignment editor software (Ibis Biosciences). All sequences obtained were compared with published sequences in the GenBank database using BLAST (Basic Local Alignment Search Tool, NCBI [http://www.ncbi.nlm.nih.gov/BLAST]).

### 2.8 Data management

Sampling was grouped into three seasons: winter-spring (Feb-Apr), summer (May-Sep), and fall-winter (Oct-Jan).

Fisher’s exact test, Pearson’s chi-squared test, and t-tests were used to analyze differences in the prevalence of shedding and in OPG between groups of animals. Results were considered significant when p  ≤ 0.05. All statistical analyses were done with STATA® (StataCorp LP, College Station, TX).

## 3 Results

### 3.1 Pre-study

*Cryptosporidium* spp. oocysts were found in 18 of the 50 (36%) samples collected in the pre-study. *Cryptosporidium* species were determined in 12 samples: 9 (75%) were *C. bovis*, 2 (17%) were *C. ryanae,* and 1 (8%) contained both *C. bovis* and *C. ryanae*. No *C. parvum* or *C. andersoni* were detected. All samples with oocysts at the detection limit (200 OPG), and 1 with 1200 OPG, had unsuccessful amplification or species determination.

Calves in single pens (1–13 days old) shed no oocysts. The highest prevalence of shedding was in the group pens with pre-weaned calves (2–13 weeks old), where 9 of 17 calves (53%) were shedding oocysts, with OPG values between 200 and 900,000. Two calves, 16 and 25 days old, shed the largest amount of *C. bovis* oocysts, with 750,000 and 900,000 OPG, respectively. In the group of young heifers (5–6 months old), 4 of 13 individuals (31%) were shedding *C. bovis* or a mix of *C. bovis* and *C. ryanae* at between 200 and 13,000 OPG. Among the two-year-old heifers, 3 out of 9 (33%) were shedding *C. ryanae* at 200–400 OPG. Among the adult cows, 2 out of 9 (22%) shed 200 OPG, with species undetermined.

### 3.2 Cross-sectional study

A total of 238 feces samples were collected; of these, 92 (38.7%) were positive for *Cryptosporidium* spp. oocysts. Species could be determined by PCR in 72 of these samples: 63 (87.5%) were *C. bovis,* 7 (9.7%) were *C. ryanae*, and 2 (2.8%) were a mix of both species. In the majority of the 20 samples where the species could not be determined, few oocysts were detected (200–800 OPG), but three such samples contained 2800–4400 OPG. No *C. parvum* or *C. andersoni* were found.

Sampled calves ranged from 1 to 63 days old (31 ± 1.10 (mean ± SEM)). Calves in the single pens ranged from 1 to 26 days old (10 ± 0.77). The calves in the indoor group pens were aged 21 to 61 days (42 ± 1.18), and those in the outdoor group pens were aged 13 to 63 days (35 ± 1.68). Each group pen held calves of approximately the same age. The smallest age difference between the youngest and oldest calf within a pen was 4 days and the largest age difference was 28 days. There was no difference in age between the two types of group pens (p = 0.759).

**Fig. 1:**
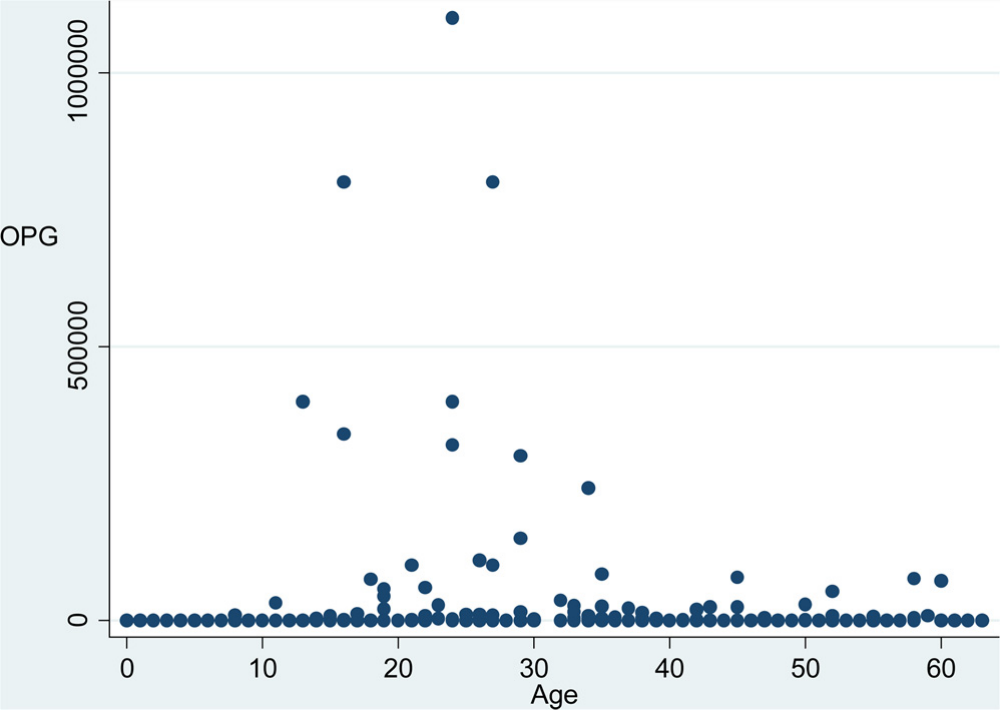
The shedding of Cryptosporidium spp. oocysts per gram feces (OPG) in calves (n = 92) of different ages (1–63 d) in a Swedish dairy herd.

The 92 calves shedding oocysts were between 2 and 61 days old (33 ± 1.48); however, the sample from the 2-day-old calf could not be species determined. *C. bovis* was identified in 65 calves from 5 to 61 days old (30 ± 1.60). *C. ryanae* was found in 9 calves from 15 to 60 days old (41 ± 7.0). The two calves that excreted both species were 4 weeks old and the OPG were 36,200 and 14,600. The mean age for non-shedding calves was 30 ± 1.53 days. The prevalence of oocyst-shedding calves was the lowest in the single pens, but there was no difference between the two types of group pens, i.e. indoors vs. outdoors (p = 0.899) (Table 1). The number of shedding calves grouped by age, without taking housing system into account, is shown in Table 2. There was a difference between calves of different age, with weeks 4 and 5 showing the largest percentage of shedding animals (53.3% and 60.0%, respectively). The OPG of the shedding calves ranged between 200 and 1.1 × 106 (geometric mean 6.88 × 103). For single infection with *C. bovis* the results ranged between 200 and 1.1 × 106 OPG (geometric mean 1.56 × 104) and for single infection with *C. ryanae* the results ranged between 200 and 1.0 × 105 OPG (geometric mean 7.53 × 103). When compared between calves of different age, the OPG was at its highest around 2–4 weeks (Fig. 1).

**Table 1:** The distribution of oocyst shedding pre-weaned calves in different housings systems in a Swedish dairy herd.

Housing	Age at sampling *days (mean)*	Total *n*	Shedding *n*	Prevalence *%*	OPG^a^
Single pen	1–26 (10)	64	13	20.3^b^	200 - 4 × 10^5^
Group pen, indoors	21–61 (42)	89	40	44.^c^	200 - 8 × 10^5^
Group pen, outdoors	13–63 (35)	74	34	45.9^c^	200 - 1.1 × 10^6^
Unknown	22–44 (35)	11	5	45.5^bc^	3 × 10^3^ - 3.2 × 10^5^
Total	1–63 (31)	238	92	38.7	200 - 1.1 × 10^6^

aOPG = oocysts per gram feces in oocyst-shedding calves. b, c = values with the same superscript are not significantly different (p > 0.05) in Fisher’s exact test.

**Table 2:** The distribution of oocyst shedding pre-weaned calves, grouped by age, in a Swedish dairy herd.

Week of life	Total *n*	Positive *n*	Prevalence *(%)*
1st	24	3	12.5^a^
2nd	27	4	14.8^a^
3rd	27	13	48.2^bc^
4th	30	16	53.3^bc^
5th	30	18	60.0^bd^
6th	32	16	50.0^bc^
7th	24	7	29.2^c^
8th	30	9	30.0^ac^
9th	14	6	42.9^acd^
Total	238	92	38.7

a–d = values with the same superscript are not significantly different (p > 0.05) in Fisher’s exact test.

Feces samples were classified as firm (37%), pasty (36%), loose (22%), and diarrheic (5%). *C. bovis* was found in 4 of the 13 (31%) feces samples that were diarrheic. No correlation was seen between OPG and diarrhea or other clinical findings such as coughing. All calves were in normal body condition. There was no difference in prevalence of shedding calves between seasons (p = 0.371). No difference was seen in shedding prevalence between female and male calves at any age (p = 0.789); however, in the single pens the females were somewhat older than the males: mean age was 12 days compared with 9 days (p = 0.028). The bedding was dry in 63% of the pens, damp in 24%, and wet in 5% (bedding condition was not recorded for 8%); no association with diarrhea or oocyst shedding were seen for this variable.

## 4 Discussion

In this repeated cross-sectional study of a Swedish dairy herd we found both *C. bovis* and *C. ryanae*, but no *C. parvum*, in the pre-weaned calves. This was not surprising, since the herd was selected because it had been positive for *C. bovis*, *C. ryanae* and *C. andersoni* in earlier samplings. This is the first long-term study focusing on the species *C. bovis* and *C. ryanae*, and it showed that the prevalence of *C. bovis/C. ryanae* was not associated with the season of the year or whether the calves were kept in indoor pens or outdoor huts.

In this context, our definition of infection dynamics includes how the infection manifests in a herd with respect to age, housing, season, etc. The earliest confirmed *C. bovis* oocyst shedding was observed in a 5-day-old calf that shed 400 OPG, and then an 8-day-old calf shedding 9000 OPG. These *C. bovis* oocysts were found earlier than the 8–10-day prepatent period reported from the only experimental infection with *C. bovis* that has been performed in calves (Fayer et al., 2005). In studies of naturally infected herds, *C. bovis* oocysts have been found in calves 7 to 9 days old (Wang et al., 2011, Silverlås and Blanco-Penedo, 2013), and these results show that the prepatent period may be shorter than previously recorded. In addition, one calf was already shedding oocysts at 2 days of age, but the oocyst count was low (200 OPG) and species was never confirmed with PCR. *C. ryanae* was first detected on day 15 and has a reported prepatent period of 11 days (Fayer et al., 2008). The calves shedding confirmed *C. bovis* oocysts had a mean age of 30 days. This is older than what was found in a New York state investigation of dairy herds, where calves shedding *C. bovis* had a mean age of 20 days (Starkey et al., 2006). The prepatent period and amount of oocysts shed vary, at least for *C. parvum,* with infection dose (Fayer et al., 2009). However, it is not known whether this is true for other *Cryptosporidium* species e.g. *C. bovis* and *C. ryanae*. In addition, the infection dose of the calves in this herd is unknown.

The prevalence of shedding calves was highest at 4 and 5 weeks of age. This peak occurred earlier than what was seen in a previous report from Sweden, where the highest prevalence of *C. bovis* was found to be in week 6 (Silverlås and Blanco-Penedo, 2013). Further, most international reports find *C. bovis* mainly in post-weaned calves (Fayer et al., 2010). In contrast, several studies of *C. parvum* have shown the highest percentages of shedding calves at weeks 1–3 (Santín et al., 2004, Fayer et al., 2007, Santín et al., 2008, Abeywardena et al., 2015). In two of these studies, which included all relevant *Cryptosporidium* species in cattle, a small peak in *C. bovis* shedding was seen at approximately 4 weeks (Santín et al., 2004, Santín et al., 2008). We found the geometric mean oocyst count to be 1.56 × 104 OPG for animals infected with *C. bovis*, and 7.53 × 103 OPG for *C. ryanae*. This is higher than in a study from New York state, which found a geometric mean for *C. bovis* of 98; however non-shedding calves were also apparently included in the mean, since the range was said to be 0 to 5.9 × 104 (Starkey et al., 2006). Our results are, however, in line with the shedding rates of 300 to > 8 × 106 OPG for *C. bovis* and 100 to 8.34 × 105 OPG for *C. ryanae* found on 40 farms in Sweden (Silverlås and Blanco-Penedo, 2013). One must bear in mind that the OPG values in the present study were calculated from single samples from each calf. The oocyst shedding follows a bell curve during infection (Zambriski et al., 2013), and it is not known at which stage of the infection the samples were taken. Also, it is likely that the sampling routine, with only one specimen examined per calf, underestimates the actual prevalence of infection (Fayer et al., 1998).

There was a difference in shedding between housing systems, with a higher prevalence in calves kept in group pens than in single pens. However, since housing was dependent on age, and there was no difference between the two group-housing systems where the calves were of similar age. The difference between single and group pens was interpreted as dependent on age, with housing as a confounder. This is in accordance with results from a study of calves up to the age of 11 months conducted on 15 U.S. farms, which found no association between housing type and infection with *Cryptosporidium* spp. (Santín et al., 2004). However, results differ between studies, and housing calves in single pens in a cow barn has been found to be associated with a higher prevalence of *Cryptosporidium* infection than housing in individual outdoor calf huts (Quigley et al., 1994). Another U.S. study comprising 3000 animals showed that calves raised outdoors or in housing with mechanical ventilation were less likely to be infected with *C. parvum* than calves that were housed indoors without ventilation (Mohammed et al., 1999). Further, the type of flooring has been identified as a significant factor in several studies, with cement flooring being protective (Castro-Hermida et al., 2002, Trotz-Williams et al., 2008). The use of an empty period between calves has been found to reduce the risk of *Cryptosporidium* infection (Maddox-Hyttel et al., 2006). In the present study, the most obvious difference between the outdoor and indoor group pens, besides location, was bedding. The indoor pens used rubber mats on concrete, with wood shavings strewn on top, whereas the outdoor pens had deep straw bedding. Furthermore, the indoor calves were in the vicinity of older heifers, while the outdoor group was not. This can be compared with results showing that calves being housed in a cow barn were at a significantly higher risk of infection with both *C. bovis* and *C. parvum* than those that were housed elsewhere (Szonyi et al., 2012). However, it is difficult to compare the effect of different housing types, since the farms included in studies generally have different types of calf pens and the time spent in each type of pen also differs (Sturdee et al., 2003, Santín et al., 2004).

No difference in the prevalence of shedding calves was detected between the different seasons: winter-spring, summer and fall-winter. Other studies of *Cryptosporidium* spp., in which the species were not specified, have revealed varying results for seasonal oocyst shedding; some showed no difference between seasons (Mohammed et al., 1999), while others saw a higher prevalence in the fall (Sturdee et al., 2003, Urie et al., 2018) or winter (Hamnes et al., 2006). A study from China that looked at two species found that *C. parvum* dominated in summer and *C. bovis* in winter (Wang et al., 2011). Sweden has marked seasonal variation in terms of temperature, precipitation, and humidity. In the Mideast of Sweden, where the study herd was located, the summer of 2012 was cool (average daily temperature around 18 °C), with high precipitation followed by a cold winter (average daily temperature around −10 °C) with a snow depth generally over 30 cm into March 2013. Spring 2013 was cooler than normal (average daily temperature around −5 °C), but with low precipitation (Swedish Meteorological and Hydrological Institute. https://www.smhi.se/en). The lack of difference in oocyst shedding between the seasons in the present study might depend on the management of the calves in this herd being very consistent over the course of the year. Also, it is difficult to assess seasonal variation when a single year is examined.

Of the 110 *Cryptosporidium*-positive samples found in the pre-study and the cross-sectional study, 84 were successfully species identified by PCR. All but 4 of the 26 samples that could not be determined by PCR all contained few oocysts (200–800 OPG); the remaining 4 contained 1200–4400 OPG. This is similar to what Silverlås (2010a) found, and the low oocyst counts—i.e. low amount of genetic material—may explain the lack of successful PCR results.

Diarrhea was seen in 5% of all the collected feces samples, which is close to the approximate average in Swedish dairy farms of 6% (personal communication Anna Duse, National Veterinary Institute). Whether or not calves were shedding *C. bovis* did not correlate with diarrhea and thus negated Silverlås’s proposition (2010a) that *C. bovis* might be pathogenic to *Cryptosporidium* naïve calves. One reason for the low pathogenicity of *C. bovis* might be that the parasite is adapted to bovines, as suggested by Starkey et al. (2006).

This report was from just one farm in Sweden, but the herd was representative in many ways. The average herd size in Sweden is 89 lactating cows —smaller than this farm’s 140 cows. However, the trend among dairy farms in Sweden is one of consolidation into fewer but larger farms (Statistics Sweden, 2018). Approximately 30% of Swedish dairy herds have an automatic milking system (Växa Sverige, 2017). The study farm was organic, and almost 15% of the total milk production in Sweden was organic in 2017 (Swedish Board of Agriculture, 2018). The biggest difference about this herd, from an international perspective, was the fact that it had for many years kept free of *C. parvum* and this presented a rare opportunity to study the dynamics of *C. bovis* and *C. ryanae.*

## 5 Conclusions

In this first longitudinal study of a herd where no *C. parvum* was present, the *C. bovis/C. ryanae* infection was most prevalent in calves aged 4–5 weeks. This is earlier than what has been found in most other studies, indicating that the infection dynamics of *C. bovis/C. ryanae* is affected by the presence of *C. parvum*. Calves’ housing type and seasonality were not associated with differences in the shedding of oocysts. Furthermore, there was no association between the presence of diarrhea and oocyst shedding.

## Conflict of interests

None.

## Funding

The study was funded by the Swedish Research Council for Environment, Agricultural Sciences and Spatial Planning (FORMAS) (grant number 221-2010-1141).
